# The Willingness for Downward Referral and Its Influencing Factors: A Cross-Sectional Study among Older Adults in Shandong, China

**DOI:** 10.3390/ijerph17010369

**Published:** 2020-01-06

**Authors:** Xiang Jing, Lingzhong Xu, Wenzhe Qin, Jiao Zhang, Lu Lu, Yali Wang, Yu Xia, An’an Jiao, Yaozu Li

**Affiliations:** 1 School of Public Health, Shandong University, Jinan 250012, China; 13209897715@163.com (X.J.); qinwenzhe09@163.com (W.Q.); 201614324@mail.sdu.edu.cn (J.Z.); lulu20170320@163.com (L.L.); wyl_0719@163.com (Y.W.); xy978742748@foxmail.com (Y.X.); jiaoanan0328@163.com (A.J.); liyaozu1211@163.com (Y.L.); 2 NHC Key Laboratory of Health Economics and Policy Research, Shandong University, Jinan 250012, China; 3 Shandong University Center for Health Economics Experiment and Public Policy Research, Jinan 250012, China

**Keywords:** hierarchical medical system, downward referral, essential medicines, China

## Abstract

*Objectives*: The aim of this study was to understand the willingness for downward referral among older adults who were hospitalized in the year before the survey and to explore its influencing factors. *Methods*: The sample was randomly selected by the multi-stage sampling method. A structural questionnaire was used to collect data from participants age 60 and above in Shandong, China, during August 2017. Data were analyzed by using descriptive statistics, one-way ANOVA, chi-square test, and multinomial logistic regression. *Results*: Of 1198 participants who were hospitalized in the year before the survey, 28.7% self-initiated downward referral, and 33.9% were willing to accept downward referral after a doctor’s advice. Multinomial logistic regression results showed that self-rated health, treatment effect in primary medical institutions, preference for outpatient service, choice of inpatient service, general understanding of essential medicines, the cost of essential medicines after zero-markup policy, and satisfaction with essential medicines’ reimbursement policy significantly correlated with older adults’ willingness for downward referral. *Conclusions*: The proportion of older adults who self-initiated downward referral was less than one-third. Doctors’ advice plays an important role in willingness for downward referral. More attention should be paid to improving the treatment effect of primary medical institutions, increasing the benefits of zero-markup policy, and ensuring a high reimbursement for the downward referral to work alongside doctors’ advice.

## 1. Introduction

The World Health Organization (WHO) considers referral an essential part of a comprehensive health care system and released documents to guide the implementation of referral systems [1], which are utilized in many countries around the world. International experiences show that a well-functioning referral system strengthens the relationship between different levels of health institutions in the healthcare system and helps to ensure people receive the most cost-effective healthcare services [2,3,4,5].

In China, there is currently a three-tier health-care system, including primary, secondary, and tertiary healthcare institutions [6]. Higher-level hospitals often have better medical resources and can provide more comprehensive health services. Referral occurs among different levels of institution according to the patient’s illness [7]. As routes designed by policy makers, patients go to primary medical institutions (PMI) for first-contact care; they are referred to secondary or tertiary hospitals if their condition is beyond the ability of the PMI (upward referral, UR), and they are referred to a lower level hospital when their illness is stable (downward referral, DR).

As early as the 1970s, China had an established effective referral system, which collapsed after the market-oriented open policy [8]. In 1997, the Chinese government re-proposed the two-way referral system, but it did not seem to achieve the original purpose of the policy [9], as research published in 2007 showed that the percentage of UR formed 96.36% of total referral, while DR only accounted for 3.64% [10]. This shows that PMIs are underutilized, whereas larger, hospital-based services (e.g., secondary and tertiary hospitals) are overused. To address these problems, in 2009, China initiated a new round of healthcare reform, which reemphasized the referral system [11], and the family doctor (FD) system was used for the implementation of referrals [12,13]. In 2015, “Guiding Opinions of the Central Committee of the CPC and the State Council on Promoting the Construction of Hierarchical Medical System” was launched to complete the referral system [14]. Unfortunately, the policy’s effect is still far from ideal [15]. Policy is one thing—practice is another. One of the major challenges is that DR is difficult [16]. For example, one study in 2014, conducted in 10 provinces in China, showed that the number of UR was five times higher that of DR [17]; another study published in 2019 found that 84.36% of patients with chronic diseases went without DR [18]. Many patients have the deeply entrenched habit of seeking treatment from large hospitals, and even if they are stable after treatment at large hospitals, they are still unwilling to be referred to lower-level hospitals.

There have been many studies on the factors that influence referral worldwide, but their focus was different. Foreign research mainly concentrated on referral from community to specialty hospitals and influencing factors that mainly include primary care physicians’ decisions [19,20], preference of patients, medical insurance, quality of care and patient satisfaction [21], patients’ socioeconomic status [22], etc. In contrast, in China, considering a patient’s freedom to choose medical institutions [23,24] and the dilemma of referring patients to lower-level hospitals, some studies focused on the patient’s DR [25,26]. For example, a study from Xu et al. deemed that the lack of trust in PMI is the main reason it is difficult to implement DR [27] with a proportion of up to 63.7% [28]. Another study, based on a system dynamics simulation model, found that higher levels of medical insurance for community healthcare systems would attract more patients to refer downwards [29]. With the exception of the above, patients’ willingness is assumed to be one of the most important factors influencing DR [30]. For most cases in China, on the patient level, DR is a risky choice. This may be attributed to the following possible reasons: the relatively simple medical equipment [31], the lack of health professionals [32], and the shortage of drugs in PMI [33]. Therefore, even if the patient meets the requirements for DR, most of them are reluctant to refer downward. Besides, drug policies potentially affect the referral decision, such as the essential medicines (EMs) system [34,35]. China introduced the National Essential Medicine Policy (NEMP) in 2009 [36], which aims at improving the affordability and the availability of EMs and increasing the service utilization in PMI. A central component of NEMP is zero-markup policy, under which drugs are sold to patients for the procurement price plus a fixed distribution cost with no profit for the sale [37]. A previous study showed that NEMP alleviated the financial burden of rural seniors and slightly improved the efficiency of primary health service utilization [38]. However, the role of EMs on the referral system is far from satisfactory due to categories of drugs [39].

At a time when the aging of populations is accelerating worldwide, China has a large number of older people. According to statistics, there were 249 million older adults aged 60 and above at the end of 2018 in China, accounting for 17.9% of the total population [40]. Undoubtedly, the aging population poses severe challenges to the healthcare system in China. Importantly, there are many facts showing that the aged are vulnerable to chronic conditions [41,42] that can be treated appropriately in PMI [43]. DR is a cost-effective choice for older adults who often have no ability to pay [44]. According to the available data, few studies focused on willingness for DR and determinants for this among older adults in China. Thus, the objectives of the present study were: (1) to investigate the willingness for downward referral among older adults who were hospitalized in the year before the survey; and (2) to explore its influencing factors.

## 2. Materials and Methods

### 2.1. Study Design and Data Collection

Our data were obtained from the 2017 survey of the Elderly Family Health Service in Shandong, China. The multi-stage random sampling was used to choose research subjects. The sampling process and the quality control measures of this survey were described in detail in a previously published paper [45]. Firstly, six counties were selected from 137 counties as the primary sampling units (PSUs) according to per capita gross domestic product (GDP) level and the geographic location of Shandong Province (which was divided into three districts and three counties that represented urban and rural areas, respectively). Secondly, 18 villages (from the rural area) and 18 communities (from suburban and urban areas) were selected as the secondary sampling units (SSUs) from each PSU. Thirdly, an average of 66 participants aged 60 or order were randomly chosen from each SSU. In total, six districts/counties and 108 villages/communities were included, and 7088 older people were selected and interviewed. Eighteen were excluded for incomplete data, meaning a total of 7070 individuals were included in the final sample. In our study, 1198 older adults, who were hospitalized in the year before the survey, were included as research objects.

All participants accepted 30 min, face-to-face interviews by trained postgraduate students from the school of public health, Shandong University. The students explained the purpose of this survey before the interview began, and a self-designed questionnaire was used to collect data. To ensure quality, completed questionnaires were checked by quality supervisors, and the Myer’s Index and the test of goodness of fit were used to evaluate quality of sample data. The Myer’s Index was estimated to be 2.19 (2.19 < 60), and the test of goodness of fit was not statistically significant (*p* > 0.05), which indicated good quality of data.

### 2.2. Dependent Variable

The willingness for DR was measured by using a situational question: “When illness is stable/much better in upper hospitals (secondary hospitals and above), are you willing to refer to lower level hospitals?” The response was divided into three categories: (a) I ask for DR (self-initiated); (b) I accept DR after doctor’s advice (doctor’s advice); (c) I have no willingness for DR (unwilling).

### 2.3. Independent Variables

Related factors regarding PMI

(1). Can you buy the drugs needed in PMI for your illness? With two response options: yes and no;
(2). How do you think of the treatment effect in PMI? With six response options: 1 = very good, 2 = good, 3 = moderate, 4 = poor, 5 = very poor, and 6 = invalid. In order to identity the associated factors, we subsequently categorized their answers into either better (ratings of good and very good) or worse (ratings of invalid, moderate, poor, and very poor).

Choice of medical institution

(1). Preference for outpatient services when ill. For ease of interpretation of the results, the response was divided into four categories: 1 = primary medical institutions (including community health systems and rural healthcare clinics); 2 = county-level medical institutions; 3 = others (including private medical institutions, traditional Chinese medical institutions, and other types); 4 = municipal level and above medical institutions;
(2). Choice of inpatient service for most recent illness. This response was also divided into four categories: 1 = primary medical institutions (e.g., community health systems); 2 = county-level medical institutions; 3 = others (including private medical institutions and other types); 4 = municipal level and above medical institutions.

Perception of EMs system

(1) Do you know the EMs system in general? (yes, no); (2) what is your opinion on the price of EMs? (high, moderate, low); (3) what is your opinion on the quality of EMs? (high, moderate, low); (4) are you satisfied with the reimbursement policy of EMs? (satisfaction, moderate, dissatisfaction); (5) do you agree with the cost-saving of EMs after zero-markup policy? (yes, no).

Socio-demographic characteristics

Socio-demographic characteristics included age, gender (male, female), residence (rural, urban), personal yearly income (four quartiles: Q1 is the poorest and Q4 is the richest), marital status (married, others), education (no education, primary school, middle school or above), numbers of non-communicable diseases (NCD), living arrangement (one, two, and three or above), self-rated health (good, fair, poor), medical insurance [urban employee basic medical insurance (UEBMI), urban and rural residents basic medical insurance (URRBMI), others, and none].

### 2.4. Statistical Analysis

All data were entered using the software Epidata 3.1, and data analysis was conducted using SPSS 24.0 (IBM Crop, Armonk, NY, USA). Firstly, the sociodemographic characteristics of older adults were analyzed with descriptive statistics and are presented in percentages. Secondly, one-way ANOVA was used to examine statistical significance in continuous variables, and chi-square test was used for categorical variables across different groups. Thirdly, in order to identify the predictors of the willingness for DR among older people, all variables were included in the multinomial logistic regression models, and willingness for DR was the independent variable, using unwilling as the reference category. The reported confidence intervals were calculated at the 95% level (95% CI). The statistical significance was 0.05.

### 2.5. Ethical Considerations

This study was approved by the ethics committee of Shandong University (No. 20170110). Informed consent for the collection and the use of information was obtained from all participants.

## 3. Results

### 3.1. Socio-Demographic Characteristics of Older Adults

Socio-demographic characteristics of older adults are presented in Table 1. Among all participants, the age range was from 60 to 94 years, and the average age was 70.44 ± 6.45, of which 714 (59.6%) were female, 917 (76.5%) lived in rural, 977 (81.6%) were married, and 466 (38.9%) had a primary degree. In addition, 945 (78.9%) with URRBM, 422 (35.2%) reported that their health was good, most of the participants (87.4%) had chronic non-communicable diseases, and 13.4% lived alone.

### 3.2. Willingness for DR

Our results showed that 344 (28.7%) were inclined to ask for DR on their own initiative, and 406 (33.9%) expressed that they would accept DR after doctor’s advice. In addition, 448 (37.4%) were unwilling to accept DR even if illness was stable/much better in the upper hospitals.

### 3.3. Univariate Analysis Results

Regarding related factors about medical institutions, 71.5% of older people believed that PMI could provide the needed drugs, and 79.2% thought that the effects of treatment in PMI were better. Up to 88% of older people stated that they go to PMI for outpatient service, and 39.5% of older people were hospitalized at the municipal level and above medical institutions for their most recent illness. In addition, regarding the perception of the EMs system, 89.3% stated that they understand little about the EMs system. Close to one-third (31.3%) of the respondents considered the price of EMs to be low, and only 1.4% believed that the quality of EMs was not good. Most older people were satisfied with the policy of EMs reimbursement (76.5%) and believed the implementation of a zero-markup policy for EMs could bring benefits for themselves (92.0%).

Details of all variables of the univariate analysis are shown in Table 1. The chi-square test showed that there were statistically significant differences in the preference for medical institutions for outpatient service ($\chi^{2}=15.234)$, choice of medical institutions for inpatient services ($\chi^{2}=15.479)$, treatment effect of PMI ($\chi^{2}=14.038)$, perceived quality of EMs ($\chi^{2}=9.667)$, satisfaction degree of the EMs reimbursement policy ($\chi^{2}=6.474),$ and cost-saving effect of EMs after zero-markup policy ($\chi^{2}=17.884)$.

### 3.4. Logistic Regression Analysis Results

The multinomial logistic regression analysis of the willingness for DR is summarized in Table 2. The analysis of self-initiated versus unwilling to accept DR showed that evaluation of the cost-saving of EMs zero markups policy had a significant effect (OR = 2.257; 95% CI = 1.130~4.510). Besides, there was a significant difference amongst the participants regarding self-rated health; the aged who had good physical health were more inclined to accept self-initiated DR (OR = 1.655; 95% CI = 1.136~2.410). Compared with older people hospitalized in municipal and above medical institutions in the year before the survey, older people who were hospitalized in PMI (OR = 1.641; 95% CI = 1.097~2.460) were more likely to ask for DR; older people who experienced a good treatment effect in PMI almost tripled their likelihood of downwards referral (OR = 1.691; 95% CI = 1.111~2.570).

The odds ratio (OR) for accepting DR after a doctor’s advice versus unwilling to accept DR showed that a preference for outpatient services (OR = 1.888; 95% CI = 1.026~3.470), satisfaction with the reimbursement policy of EMs (OR = 2.277; 95% CI = 1.192~4.350), and general understanding of EMs (OR = 1.636; 95% CI = 1.027~2.610) were the determinants linked with willingness for DR among older people.

## 4. Discussion

Our study found that only 28.7% of older adults who were hospitalized in the year before the survey were willing to self-initiate DR. It is noteworthy that 33.9% were willing to accept DR after a doctor’s advice. Due to the asymmetrical information between patients and doctors [46], patients rely more on the doctor’s advice when making medical decisions. However, one study found that only 20.8% of doctors in tertiary hospitals are willing to recommend DR when the patient meets the criteria for referral [30]. Thus, there is an urgent need to establish an incentive mechanism and formulate a unified referral standard to encourage and guide doctors’ referral behaviors. In addition, 37.4% of patients were still unwilling to accept DR, which is slightly lower than the research in Xuzhou city by Gao et al. [47] and in Dalian by Liu et al. [48] but close to the result in Guangzhou city by Zhou et al. (37.46%) [18] and higher than that in Changsha city by Wang et al. (22.4%) [49]. Besides, our study further identified some other factors as being significantly associated with willingness for DR: treatment effect of PMI, self-rated health, general understanding of EMs, evaluation of the cost-saving of effect EMs zero-markup policy, and satisfaction with EMs reimbursement policy.

Compared with people who were unwilling to accept DR, older people who evaluated a better treatment effect in PMI were more likely to ask for DR. This is consistent with previous research that found that the medical level of PMI is an important factor affecting the choice of patients seeking medical treatment [50]. As medical institutions receiving patients from higher-level hospitals, PMI must be able to provide a follow-up healthcare service for patients. To date, China has made remarkable progress in its primary healthcare system, as our results showed that most older adults (79.2%) evaluated that the treatment effect in PMI is better. However, there is still much room for improvement [51]. Therefore, at the governmental level, more policies should be made to strengthen the capabilities of PMI and increase government investment in PMI. Strengthening social propaganda for PMI is essential. Meanwhile, PMI should focus on providing a follow-up service, rehabilitation guidance, and appointment referral for older adults, as well as establishing a long-term patient–physician relationship through the general practitioner contract service to enhance patients’ sense of fulfillment and satisfaction for PMI.

In addition, self-rated health exerts a statistically significant effect on willingness for DR, and older people who had good self-rated health were more likely to request DR on their own initiative. Self-rated health can reflect the physical and the mental health status of individuals comprehensively [52], and former studies showed that self-rated health can effectively predict mortality [53] and quality of life, especially among older people [54]. When faced with a referral choice, the aged who rated good physical health tended to believe that the medical services of lower-level hospitals can meet their medical needs. The result makes sense, because patients usually choose medical institutions according to their physical condition [55]. Therefore, we suggest that, on the one hand, doctors need to explain patients’ conditions to them in detail, and, on the other hand, hospitals should pay attention to regulating the negative emotions of hospitalized patients.

Our study also found that some factors regarding the perception of EMs were significantly associated with the willingness for DR. NEMP aims to reduce excessive unnecessary spending. To date, China has implemented the NEMP [56,57], and the high cost of medical services for the public has been relieved [58]. Several studies have proved that the EMs system had a positive effect in reducing both outpatient and inpatient expenses at the grassroots level [59,60], and then more patients intended to return to PMI [61].

In our study, using the groups who were unwilling to accept DR as the reference, the general understanding of EMs, the evaluation of the cost-saving effect of EMs after the zero-markup policy, and the satisfaction with the reimbursement policy of EMs were significant factors influencing the willingness for DR. These findings revealed that EMs have a significant effect on the referral system to some extent. On the one hand, the aged who deemed that implementing a zero-markup policy for EMs can bring benefits were more willing to ask for DR (125.7% higher odds of willingness for DR). Medicines are sold with no markup from wholesale to retail price, which makes patients directly feel “visible and tangible” benefits, meaning they are more likely to go to PMI for needed drugs [62]. On the other hand, moderate satisfaction with the reimbursement policy of EMs was also significantly associated, with 127.7% higher odds of willingness for DR compared with dissatisfaction. Policy stipulates that the reimbursement ratio of EMs is higher than that of non-essential medicines. Our study found that older adults were generally satisfied with the reimbursement policy of EMs, and the proportion was 76.5%, indicating that the implementation of the EMs system has indeed reduced their medical burden. However, it is undeniable that sometimes PMI cannot fully meet the needs of patients, being constrained by the essential medicines list (EML). This may hinder the implementation of DR [63,64]. Considering this point, one study concluded that the implementation of DR was difficult in the context of NEMP [34]. Another study considered that the insufficient availability of EMs prevents PMI from meeting the drug needs of some patients, thus affecting willingness for DR [39]. Therefore, we suggest that EML should be adjusted by need, and PMI should be equipped with non-essential drugs for patients. At the same time, efforts should be made to gain the benefits of the zero-markup policy and high reimbursement for the DR, and policies should be formulated to increase the subsidies for PMI.

Similar to another study, our findings found that preferences towards medical institutions have a significant influence on the willingness for DR [65]. Older adults who were hospitalized in PMI for the last time tended to ask for DR, and those choosing PMI for outpatient services tended to accept DR after a doctor’s advice. A possible explanation might be assumed in older people’s trust in PMI. Based on the experience seeking medical treatment, they realize that the capability of PMI can meet their medical needs. This also means encouraging first-contact care in PMI is essential for the implementation of DR.

According to the available data, no study has analyzed willingness for DR by dividing the responses into three categories: willingness for self-initiated DR, willingness to accept DR after a doctor’s advice, and unwillingness. In our study, we classified the responses in this way to gain more comprehensive information. Meanwhile, it is undeniable that there are several limitations in this study. Firstly, our study was a cross-sectional design, and the relationship between the influencing factors and the willingness for DR cannot be interpreted as cause and effect. Secondly, personal information was self-reported, including the experience of medical care, which leads to the possibility of subjective bias. Thirdly, it is possible that social desirability may have affected the responses, considering that the participants were not hospitalized at the time of the study.

## 5. Conclusions

Our study found that less than one-third of older adults who were hospitalized in the year before the survey asked for DR, while more than one-third would accept DR after a doctor’s advice. There are various influencing factors on willingness for DR, including self-reported health, choice of medical institution, treatment effect of PMI, general understanding of EMs, evaluation of the cost-saving effect of EMs after the zero-markup policy, and satisfaction with the reimbursement policy of EMs. Based on the above results, we suggest that more attention should be paid to improving the treatment effect of PMI, gaining more benefits from the zero-markup policy and a high reimbursement for the downward referral, to work alongside the role of doctors’ advice. In sum, more time and effort are required for the referral system to be improved, and personal willingness cannot be changed overnight.

## Figures and Tables

**Table 1:** Factors associated with older adults’ willingness for downward referral (DR).

Observations	Willingness of DR			Total	$\chi^{2}/F$	*p*-Value
	Self-Initiated	Doctor’s Advice	Unwilling	*n* (%)		
	344 (28.7)	406 (33.9)	448 (37.4)	1198		
**Panel 1 Socio-Demographics**						
Age mean ± SD	70.67 ± 6.37	70.52 ± 6.56	70.19 ± 6.42	70.44 ± 6.45	0.599 ^a^	0.550
Gender						
Male	150 (31.0)	159 (32.9)	175 (36.2)	484 (40.4)	2.058 ^b^	0.357
Female	194 (27.2)	247 (34.6)	273 (38.2)	714 (59.6)		
Residence						
Rural	289 (31.5)	304 (33.2)	324 (35.3)	917 (76.5)	16.546 ^b^	<0.001
Urban	55 (19.6)	102 (36.3)	124 (44.1)	281 (23.5)		
Income *						
Q1	101 (32.9)	91 (29.6)	115 (37.5)	307 (25.6)	13.757 ^b^	0.032
Q2	90 (30.1)	104 (34.8)	105 (35.1)	299 (25.0)		
Q3	89 (30.4)	104 (35.5)	100 (34.1)	293 (24.5)		
Q4	64 (21.4)	107 (35.8)	128 (42.8)	299 (25.0)		
Marital status						
Others ^#^	56 (25.3)	91 (41.2)	74 (33.5)	221 (18.4)	6.429 ^b^	0.040
Married	288 (29.5)	315 (32.2)	374 (38.3)	977 (81.6)		
Education						
No education	117 (29.2)	149 (37.2)	135 (33.7)	401 (33.5)	6.002 ^b^	0.199
Primary	136 (29.2)	156 (33.5)	174 (37.3)	466 (38.9)		
Junior or above	91 (27.5)	101 (30.5)	139 (42.0)	331 (27.6)		
Medical insurance						
UEBMI	57 (24.1)	70 (29.5)	110 (46.4)	237 (19.8)	10.504 ^b^	0.033
URRBMI	283 (29.9)	330 (34.9)	332 (35.1)	945 (78.9)		
Others and none	4 (25.0)	6 (37.5)	6 (37.5)	16 (1.3)		
Number of NCD						
None	51 (33.8)	52 (34.4)	48 (31.8)	151 (12.6)	4.043 ^b^	0.671
One	106 (28.1)	125 (33.2)	146 (38.7)	377 (31.5)		
Two	114 (28.7)	130 (32.7)	153 (38.5)	397 (33.1)		
Three or above	73 (26.7)	99 (36.3)	101 (37.0)	273 (22.8)		
Self-rated health						
Good	143 (33.9)	134 (31.8)	145 (34.4)	422 (35.2)	9.702 ^b^	0.046
Fair	99 (25.6)	142 (36.8)	145 (37.6)	386 (32.2)		
Poor	102 (26.2)	130 (33.3)	158 (40.5)	390 (32.6)		
Living arrangement						
1	33 (20.5)	76 (47.2)	52 (32.3)	161 (13.4)	17.784 ^b^	0.001
2	244 (29.9)	269 (32.9)	304 (37.2)	817 (68.2)		
3~	67 (30.5)	61 (27.7)	92 (41.8)	220 (18.4)		
**Panel 2 Related Factors Regarding PMI**						
Drug needed for your illness in PMI						
No	92 (26.9)	125 (36.5)	125 (36.5)	342 (28.5)	1.639 ^b^	0.441
Yes	252 (29.4)	281 (32.8)	323 (37.7)	856 (71.5)		
Treatment effect of PMI						
Better	296 (31.2)	314 (33.1)	339 (35.7)	949 (79.2)	14.038 ^b^	0.001
Worse	48 (19.3)	92 (36.9)	109 (43.8)	249 (20.8)		
**Panel 3 Choice of Medical Institution**						
Preference for outpatient services when ill						
Primary	311 (29.5)	370 (35.1)	373 (35.4)	1054 (88)	15.234 ^b^	0.019
County-level	9 (23.1)	10 (25.6)	20 (51.3)	39 (3.3)		
Multiple level and above	20 (23.3)	21 (24.4)	45 (52.3)	86 (7.2)		
Others	4 (21.1)	5 (26.3)	10 (52.6)	19 (1.6)		
Choice of inpatient service for most recent illness						
Primary	120 (34.9)	116 (33.7)	108 (31.4)	344 (28.7)	15.479 ^b^	0.017
County-level	102 (29.1)	120 (34.3)	128 (36.6)	350 (29.2)		
Multiple level and above	114 (24.1)	162 (34.2)	197 (41.6)	473 (39.5)		
Others	8 (25.8)	8 (25.8)	15 (48.4)	31 (2.6)		
**Panel 4 Perception of EMs**						
General understanding of EMs						
Yes	36 (28.1)	51 (39.8)	41 (32.0)	128 (10.7)	2.619 ^b^	0.270
No	308 (28.8)	355 (33.2)	407 (38)	1070 (89.3)		
Perceived price on EMs						
High	107 (30.0)	106 (29.7)	144 (40.3)	357 (29.8)	0.384 ^b^	0.535
Moderate	132 (28.3)	160 (34.3)	174 (37.3)	466 (38.9)		
Low	105 (28)	140 (37.3)	130 (34.7)	375 (31.3)		
Perceived quality of EMs						
Good	274 (30.3)	314 (34.8)	315 (34.9)	903 (75.4)	9.667 ^b^	0.002
Moderate	68 (24.5)	84 (30.2)	126 (45.3)	278 (23.2)		
Poor	2 (11.8)	8 (47.1)	7 (41.2)	17 (1.4)		
Satisfaction with EMs reimbursement policy						
Satisfaction	279 (30.4)	302 (32.9)	336 (36.6)	917 (76.5)	6.474 ^b^	0.011
Moderate	42 (23.7)	77 (43.5)	58 (32.8)	177 (14.8)		
Dissatisfaction	23 (22.1)	27 (26)	54 (51.9)	104 (8.7)		
Cost-saving effect of EMs after zero-markup policy						
Yes	327 (29.7)	382 (34.7)	393 (35.7)	1102 (92.0)	17.884 ^b^	<0.001
No	17 (17.7)	24 (25.0)	55 (57.3)	96 (8.0)		

* Income: personal yearly income; ^#^ Others: not married/divorced/widowed or others; SD: standard deviation; ^a^ one-way AVOVA; ^b^ chi-square test; UEBMI: urban employee basic medical insurance; URRBMI: urban and rural residents basic medical insurance; PMI: primary medical institutions; EMs: essential medicines.

**Table 2:** The multinomial logistic regression analysis of older people’s willingness for DR.

Observations	Self-Initiated vs. Unwilling		Doctor’s Advice vs. Unwilling	
	β	OR	β	OR
**Panel 1 Socio-Demographics**				
Age	0.015	1.015 (0.990~1.040)	0.001	1.001 (0.978~1.026)
Gender (Ref: Female)				
Male	0.079	1.082 (0.779~1.510)	0.133	1.142 (0.831~1.570)
Residence (Ref: Urban)				
Rural	0.482	1.620 (0.883~2.970)	−0.477	0.621 (0.362~1.070)
Income (Ref: Q4)				
Q1	0.328	1.388 (0.790~2.440)	−0.432	0.649 (0.386~1.090)
Q2	0.162	1.176 (0.668~2.070)	−0.239	0.787 (0.470~1.320)
Q3	0.346	1.413 (0.822~2.430)	−0.202	0.817 (0.501~1.330)
Marital status (Ref: Married)				
Others	0.367	1.443 (0.753~2.770)	0.071	1.074 (0.556~2.080)
Education (Ref: Junior or above)				
No education	−0.095	0.910 (0.578~1.430)	0.404	1.498 (0.964~2.330)
Primary	−0.018	0.982 (0.667~1.450)	0.128	1.137 (0.781~1.660)
Medical insurance (Ref: Others and none)				
UEBMI	0.049	1.051 (0.256~4.310)	−0.836	0.433 (0.120~1.560)
URRBMI	−0.029	0.972 (0.256~3.690)	−0.091	0.913 (0.276~3.020)
Number of NCD (Ref: Three and above)				
None	0.200	1.221 (0.713~2.090)	0.132	1.141 (0.679~1.920)
One	−0.059	0.942 (0.621~1.430)	−0.115	0.892 (0.603~1.320)
Two	−0.043	0.957 (0.637~1.440)	−0.177	0.838 (0.571~1.230)
Self-rated health (Ref: Poor)				
Good	0.504	1.655 (1.136~2.410) **	0.151	1.163 (0.806~1.680)
Fair	0.125	1.134 (0.779~1.650)	0.267	1.306 (0.921~1.850)
Living arrangement (Ref: ≥ 3)				
1	−0.534	0.586 (0.281~1.220)	0.622	1.862 (0.925~3.750)
2	0.143	1.153 (0.773~1.720)	0.215	1.240 (0.830~1.850)
**Panel 2 Related Factors Regarding PMI**				
Drug needed for your illness in PMI (Ref: Yes)				
No	0.330	1.391 (0.982~1.970)	0.323	1.382 (0.994~1.920)
Treatment effect of PMI (Ref: Worse)				
Better	0.525	1.691 (1.111~2.570) *	−0.085	0.919 (0.633~1.330)
**Panel 3 Choice of Medical Institution**				
Preference for outpatient services when ill (ref: multiple and above)				
Primary	0.091	1.096 (0.584~2.050)	0.635	1.888 (1.026~3.470) *
County-level	−0.216	0.806 (0.292~2.230)	0.154	1.166 (0.435~3.120)
Others	−0.295	0.744 (0.196~2.820)	0.054	1.055 (0.302~3.690)
Choice of inpatient service for most recent illness (Ref: Multiple and above)				
Primary	0.495	1.641 (1.097~2.460) *	0.263	1.300 (0.876~1.930)
County-level	0.185	1.203 (0.816~1.770)	0.11	1.116 (0.771~1.620)
Others	−0.055	0.947 (0.371~2.420)	−0.278	0.757 (0.299~1.920)
**Panel 4 Perception of EMs**				
General understanding of EMs (Ref: No)				
Yes	0.263	1.301 (0.785~2.160)	0.492	1.636 (1.027~2.610) *
Perceived price of EMs (Ref: Low)				
High	0.162	1.176 (0.801~1.730)	−0.217	0.805 (0.556~1.170)
Moderate	0.020	1.021 (0.705~1.480)	−0.192	0.826 (0.583~1.170)
Perceived quality of EMs (Ref: Poor)				
Good	0.750	2.118 (0.414~10.800)	−0.066	0.936 (0.311~2.810)
Moderate	0.568	1.765 (0.341~9.140)	−0.513	0.599 (0.196~1.830)
Satisfaction with EMs reimbursement policy (Ref: Dissatisfaction)				
Satisfaction	0.229	1.257 (0.669~2.360)	0.176	1.192 (0.659~2.160)
Moderate	0.392	1.481 (0.729~3.010)	0.823	2.277 (1.192~4.350) *
Cost-saving effect of EMs after zero-markup policy (Ref: No)				
Yes	0.814	2.257 (1.130~4.510) *	0.596	1.816 (0.979~3.370)

** *p* < 0.01; * *p* < 0.05; “ref” refers to the reference group; β: regression coefficient; OR: odds ratio; CI: confidence interval.

## Abbreviations

EMs	essential medicines
EML	essential medicines list
PMI	primary medical institutions
UEBMI	Urban Employee Basic Medical Insurance
URRBMI	Urban and Rural Residents Basic Medical Insurance
NCD	non-communicable diseases
DR	downward referral
UR	upward referral
NEMP	National Essential Medicines Policy

## References

[1] WHO Management of Health Facilities: Referral Systems. https://www.who.int/management/facility/referral/en/.

[2] Liddy C., Arbab-Tafti S., Moroz I., Keely E. (2017). Primary care physician referral patterns in Ontario, Canada: A descriptive analysis of self-reported referral data. BMC Fam. Pract..

[3] Kim J., Mersereau J.E. (2015). Early referral makes the decision-making about fertility preservation easier: A pilot survey study of young female cancer survivors. Support. Care Cancer.

[4] Lei H., Bian Y., Liu X., Li S. (1996). Investigation on referral of outpatients and inpatients in tertiary general hospitals and its economic significance. Soft Sci. Health.

[5] Bai G., Zhou Y., Luo L., Wang Z. (2019). Resource allocation for chronic diseases based on a patient willingness survey. Int. J. Health Plan. Manag..

[6] Eggleston K., Ling L., Meng Q.Y., Lindelow M., Wagstaff A. (2008). Health service delivery in China: A literature review. Health Econ..

[7] Lu C., Zhang Z., Lan X. (2019). Impact of China’s referral reform on the equity and spatial accessibility of healthcare resources: A case study of Beijing. Soc. Sci. Med..

[8] Wang Q. (2016). The Analysis on Operational Mechanisms of Tiered Health Care System: A Case study Based on Xiamen. Master’s Thesis.

[9] Liu M., Chen J., Peng X. (2004). Problems Existing in Community Health Center Carrying out Two-way Referral and Countermeasures. Chin. Health Serv. Manag..

[10] Wan B., Liu S., Feng X. (2007). Analysis of existing problems and solutions of the implementation of two-way referral in Changchun. Med. Soc..

[11] Chen Z. (2009). Launch of the health-care reform plan in China. Lancet.

[12] Shang X., Huang Y., Li B.E., Yang Q., Zhao Y., Wang W., Liu Y., Lin J., Hu C., Qiu Y. (2019). Residents’ Awareness of Family Doctor Contract Services, Status of Contract with a Family Doctor, and Contract Service Needs in Zhejiang Province, China: A Cross-Sectional Study. Int. J. Environ. Res. Public Health.

[13] Opinions on Deepening the Reform of the Medical and Health System. http://www.gov.cn/jrzg/2009-04/06/content_1278721.htm.

[14] Guiding Opinions of the Central Committee of the CPC and the State Council on Promoting the Construction of Hierarchical Medical System. http://www.gov.cn/zhengce/content/2015-09/11/content_10158.htm.

[15] Zuo X., Zhen C., Ye X., Song H., Wang T., Guan Z., Meng K. (2018). The current situation and effectiveness evaluation of medical staff’s participation in medical alliances in Beijing. Chin. J. Health Policy.

[16] Gao K., Gan X. (2015). Behavioral Decision in Two-way Referral and Its Influencing Factors in China. Chin. Gen. Pract..

[17] Zhang M., Ding X., Gao Y. (2016). An Investigation of Medical and Public Health Services of Community Health Centers. Chin. Health Serv. Manag..

[18] Zhou R., Cui F., Yao W. (2019). Study on Willingness and Influencing Factors of Two-Way Referral for Patients with Chronic Diseases. Med. Soc..

[19] Mehrotra A., Forrest C.B., Lin C.Y. (2011). Dropping the Baton: Specialty Referrals in the United States. Milbank Q..

[20] Tandjung R., Morell S., Hanhart A., Haefeli A., Valeri F., Rosemann T., Senn O. (2017). Referral determinants in Swiss primary care with a special focus on managed care. PLoS ONE.

[21] Greenwald L., Cromwell J., Adamache W., Bernard S., Drozd E., Root E., Devers K. (2006). Specialty versus community hospitals: Referrals, quality, and community benefits. Health Aff..

[22] Sørensen T.H., Olsen K.R., Vedsted P. (2009). Association between general practice referral rates and patients’ socioeconomic status and access to specialised health care: A population-based nationwide study. Health Policy.

[23] Wu D., Lam T.P., Lam K.F., Zhou X.D., Sun K.S. (2017). Health reforms in china: The public’s choices for first-contact care in urban areas. Fam. Pract..

[24] Wu D., Lam T.P. (2016). Underuse of Primary Care in China: The Scale, Causes, and Solutions. J. Am. Board Fam. Med..

[25] Zhao Y., Zhang H., Zhu L., Zhao X. (2017). Analysis of willingness and influencing factors of downward referral of patients in Tangshan. Manag. Obs..

[26] Gan X., Jia Q., Li H. (2011). Influential Factors to Downward Referral for Residents in Rural Areas. Chin. Gen. Pract..

[27] Xu C.N., Quan S.C., Zhou X.Z., Wang R.J., Wen J.M., Pan J.Y. (2009). Quantitative Analysis on Influencing Factors of Being Difficult in Two-Way Referral Mode. Chin. Health Serv. Manag..

[28] Wang H., Feng Z., Li P. (2012). Analysis of influencing factors of referral from hospital to comunity in two-way referral system. Chin. J. Hosp. Adm..

[29] Li M., Zhang Y., Lu Y., Yu W., Nong X., Zhang L. (2018). Factors influencing two-way referral between hospitals and the community in China: A system dynamics simulation model. Simul. Trans. Soc. Model. Simul. Int..

[30] Yu W., Li M., Nong X., Ding T., Ye F., Liu J., Dai Z., Zhang L. (2017). Practices and attitudes of doctors and patients to downward referral in Shanghai, China. BMJ Open.

[31] Bhattacharyya O., Yin D., Wong S.T., Chen B. (2011). Evolution of primary care in China 1997–2009. Health Policy.

[32] Zhao Y., Li X., Zhang Y. (2010). Status and Effect of Two-way Referral System in China: A systematic review. Chin. Gen. Pract..

[33] Shen Y., Huang W., Ji S., Yu J., Li M. (2018). Efficacy, Problems and Current Status of Two-way Referral from 1997 to 2017 in China: A Systematic Review. Chin. Gen. Pract..

[34] Qin Y., Wu Z., Lu J. (2012). Two-way Referral Based on the Community Doctors’ Behaviors Under the Background of the National Essential Drugs System: An Analysis of the Status and Its Strategy. Chin. Gen. Pract..

[35] Lin X. (2013). Impact of Essential Medicines on the Two-Way Referral and Policy Making in Chongqing under the New Health Reformation. Master’s Thesis.

[36] Council T.S. Implementation Plan for the Recent Priorities of the Health Care System Reform (2009–2011). http://www.gov.cn/zwgk/2009-04/07/content_1279256.htm.

[37] Cheng W., Fang Y., Fan D., Sun J., Shi X., Li J. (2012). The effect of implementing “medicines zero mark-up policy” in Beijing community health facilities. South. Med. Rev..

[38] Wang Y., Zhu Y., Shi H., Sun X., Chen N., Li X. (2019). The Effect of the Full Coverage of Essential Medicines Policy on Utilization and Accessibility of Primary Healthcare Service for Rural Seniors: A Time Series Study in Qidong, China. Int. J. Environ. Res. Public Health.

[39] Lin X., Feng Z., Zhang H., Feng J. (2012). Improving the availability of basic pharmaceuticals to promote the development of Two-Way Referral System in Communities. Chin. Health Serv. Manag..

[40] National Bureau of Statistics of the People’s China Statistical Bulletin of National Economic and Social Development in 2018. http://www.gov.cn/xinwen/2019-02/28/content_5369270.htm.

[41] WHO World Report on Ageing and Health. https://www.who.int/ageing/publications/world-report-2015/en/.

[42] Cui J., Mao F., Wang Z. (2016). Comorbidity of common chronic diseases among the elderly in China. Chin. J. Public Health.

[43] Niu Y.D., Ye T., Zhang Y., Zhang L. (2019). Can Primary Medical Institutions Lead to Worse Health Status for Patients with Noncommunicable Diseases Compared with High-Level Hospitals? A Follow-Up Observation Study in China. Int. J. Environ. Res. Public Health.

[44] Luo J.H., Zhang X.L., Jin C.G., Wang D.M. (2009). Inequality of access to health care among the urban elderly in northwestern China. Health Policy.

[45] Zhang J., Xu L.Z., Li J.J., Sun L., Ding G., Qin W.Z., Wang Q., Zhu J., Yu Z.H., Xie S. (2018). Loneliness and Health Service Utilization among the Rural Elderly in Shandong, China: A Cross-Sectional Study. Int. J. Environ. Res. Public Health.

[46] Fe E., Powell-Jackson T., Yip W. (2017). Doctor Competence and the Demand for Healthcare: Evidence from Rural China. Health Econ..

[47] Gao X., Zhang X.Y., Li S. (2017). Willingness of Down-referral of Community Residents and Its Influencing Factors. Chin. Gen. Pract..

[48] Liu Y., Jiang J., Sha L. Willingness of Dual Referral of Elderl Y Patients and Its Influencing Factors in Chongqing Medicine. http://kns.cnki.net/kcms/detail/50.1097.r.20191204.1702.030.html.

[49] Wang X., Yan X., Wei X., Fan X., Liu Q., Guo Y. (2018). Exploration on impacting factors for restricting two-way referral system from patient’s perspective. Chongqing Med..

[50] Xu J., Qian D., Zhang D. (2015). Types of Diseases Suitable for Downward Referral From Hospitals to Community Health Centers. Chin. Gen. Pract..

[51] Li X., Lu J., Hu S., Cheng K.K., De Maeseneer J., Meng Q., Mossialos E., Xu D.R., Yip W., Zhang H. (2017). The primary health-care system in China. Lancet.

[52] Wu S., Wang R., Zhao Y., Ma X., Wu M., Yan X., He J. (2013). The relationship between self-rated health and objective health status: A population-based study. BMC Public Health.

[53] Kaplan G.A., Goldberg D.E., Everson S.A., Cohen R.D., Salonen R., Tuomilehto J., Salonen J. (1996). Perceived health status and morbidity and mortality: Evidence from the Kuopio Ischaemic Heart Disease Risk Factor Study. Int. J. Epidemiol..

[54] Damian J., Ruigomez A., Pastor V., Martin-Moreno J.M. (1999). Determinants of self assessed health among Spanish older people living at home. J. Epidemiol. Community Health.

[55] Liu L., Liu C., Duan Z., Pan J., Yang M. (2019). Factors associated with the inter-facility transfer of inpatients in Sichuan province, China. BMC Health Serv. Res..

[56] Yin S., Song Y., Bian Y. (2014). Does the Essential Medicines Policy Succeed in China? Empirical Study on Rational Medicine Use in Primary Health Care Institutions. Ther. Innov. Regul. Sci..

[57] He J., Tang M., Ye Z., Jiang X., Chen D., Song P., Jin C. (2018). China issues the National Essential Medicines List (2018 edition): Background, differences from previous editions, and potential issues. Biosci. Trends.

[58] Wang D. (2014). Study on the Impact of National Essential Medicine System on Medical Expenses of the Patients with Medical Insurance in Our Hospital. China Pharm..

[59] Li Q., Chen F., Yang M., Lu L.Y., Pan J., Li X.S., Meng Q. (2018). The Effect of China’s National Essential Medicine Policy on Health Expenses: Evidence From a National Study. Inq. J. Health Care Organ. Provis. Financ..

[60] Zhang X., Wu Q.H., Liu G.X., Li Y., Gao L.J., Guo B., Fu W.Q., Hao Y.H., Cui Y., Huang W.D. (2014). The effect of the National Essential Medicines Policy on health expenditures and service delivery in Chinese township health centres: Evidence from a longitudinal study. BMJ Open.

[61] Guo Z.G., Guan X.D., Shi L.W. (2017). The impacts of implementation of National Essential Medicines Policies on primary healthcare institutions: A cross-sectional study in China. BMC Health Serv. Res..

[62] Yu W., Hu X. (2014). Changes of Medication Behavior of Community Residents after the Implementation of Zero Rate Policy. Pharm. Clin. Res..

[63] Lei M., Feng Z. (2011). Impact of National Essential Medicine Policy on Two-Way Referral System and the Countermeasures. Chin. Gen. Pract..

[64] Zhao Y., Yin W., Hu J., Guo H. (2014). Evaluation the Policy Effects of National Essential Medicine System from the Perspective of Hospitalized Patients. Chin. Health Serv. Manag..

[65] Shan N., Li J. (2014). Analysis of Downward Referral Will of Patients in a Secondary Hospital, Beijing. Med. Soc..

